# Dislocation of the Femur into the Perinæum

**Published:** 1852-07

**Authors:** 


*Dislocation of the Femur into the Perinæum.* By Professors PARKER and POPE.

The following case was reported to the New York Medical Gazette, in 1841, by Prof. Parker, in whose practice it occurred; and being an entirely new form of luxation of this bone, we have thought it worthy of a republication in this connection.

The patient, Mr. E——, aged about thirty-five, was a caulker by occupation, and the accident happened while he was at work under the bottom of a canal boat, July 20th, 1831. The boat was raised upon props three and a half feet long. He was standing, bent forward very much, and his feet far astride. Between his feet there was lying a piece of round timber, a foot in diameter. While at work in this position, the props gave way, the boat came down, killing one of the workmen, and forcing the patient down by the side of the timber over which he was standing, in such a manner that the left thigh was placed between it and the bottom of the boat. On being extricated from this situation, the left limb was found standing at a right angle with the trunk, the toes were turned a little inward, the natural form of the nates lost, and the head of the bone distinctly felt in rotations in the perineum behind the scrotum, and near the bulb of the urethra.

Reduction was effected without much difficulty in the following manner: The patient was placed upon a table, resting upon his back, and the pelvis confined by passing a strip of muslin around it. Extension was then made downward and outward accompanied by moderate rotation, and in this way the head of the bone was made to surmount the ramus of the ischium, and to pass into the foramen thyroideum, changing the luxation from a perineal to a thyroideum. From this position, the bone was replaced in the acetabulum, by carrying the luxated limb strongly across the sound one. The patient soon recovered the use of the joint.

The only example of the luxation of the femur in perineo, to which we can refer, in addition to the one here recorded, was reported to the St. Louis Medical and Surgical Journal, July, 1850, by Prof. Charles A. Pope, of the St. Louis University. It is the following:

"J. B., an Irishman, aged forty, on entering the St. Louis Hospital, gave the following account of his accident, which had occurred six hours previously: He was engaged in excavating earth, and having undermined a considerable band, it unexpectedly fell upon his back, catching him in a bent position, with his legs stretched widely asunder. The weight crushed him to the earth, simultaneously fracturing both bones of his right leg, the lower extremity of the radius of the same side, and also dislocating the left hip. On examination, the fractures were readily recognized, and my attention was at once directed to the peculiar position of the left thigh. This was thrown quite at a right angle to the body, and somewhat forward. The normal site of the great trochanter offered a cavity in which the fist could easily be placed, while the head of the bone was both seen and felt below the skin, and raising the raphi of the perineum. On rotation, which was difficult, and caused intense suffering, the movements of the *caput femoris* were distinctly visible. There was experienced by the patient an inability of voiding urine, which was, doubtless, produced by the pressure of the head of the bone upon the urethra, and the difficulty continuing after reduction, relief was afforded only by the use of the catheter. This trouble soon subsided. The fractures having been attended to, and the patient put under the full effect of chloroform, two loops were applied, interlocking each other in the groin, and using the leg as a lever, extension, by means of the pulleys, was made transversely to the axis of the body. A steady force was kept up for a short time, and the thigh-bone glided into its socket with a snap that was heard by every attendant and patient in the large ward, where, for the convenience of the pillars, the operation was performed. The patient, on recovering from the influence of the anæsthetic, seemed much delighted, and said that he had felt no pain whatever." Dr. Pope continues: "It might be supposed that there may have been, in these instances, some error, and that the displacement of the head was merely into the foramen ovale, and not into the perineum. I feel confident, however, that both (referring to a verbal report of Dr. Parker's case) were really dislocations, as reported. The various medical gentlemen who examined the case with me, all coincided in my opinion. The direction of the displacing force in these two varieties of luxation is precisely the same, and but little greater violence would be required to throw the head on, past the foramen ovale, and branches of the ischium and pubis, into the perineum. In the cases cited, there was the same position of the body, and of the outstretched legs, and the attendant cause and circumstances were just such as would most likely favor the production of such an accident. Had the head of the femur, in the case of J. B., been situated in the foramen ovale, it could not have been so plainly seen and felt, covered as it would have been, by the thickness of the interposed muscular attachments. Besides, the exaggerated position of the thigh, and the direct pressure of the head of the bone on the urethra, causing a retention of urine, are, to my mind, conclusive evidence of the nature of the case; for although, in the latter instance, some irritation might be propagated from the head, displaced in the foramen ovale, yet it would scarcely be so great as to cause retention of urine to the extent observed in the case above related."—*New York Jour. of Med.*

